# Inhibition of the RNA polymerase III-mediated dsDNA-sensing pathway of innate immunity by vaccinia virus protein E3

**DOI:** 10.1099/vir.0.021998-0

**Published:** 2010-09

**Authors:** Robert Valentine, Geoffrey L. Smith

**Affiliations:** Section of Virology, Faculty of Medicine, Imperial College London, St Mary's Campus, Norfolk Place, London W2 1PG, UK

## Abstract

The vaccinia virus E3 protein is an important intracellular modulator of innate immunity that can be split into distinct halves. The C terminus contains a well defined dsRNA-binding domain, whereas the N terminus contains a Z-DNA-binding domain, and both domains are required for virulence. In this study, we investigated whether the E3 Z-DNA-binding domain functions by sequestering cytoplasmic dsDNA thereby preventing the induction of type I interferon (IFN). In line with this hypothesis, expression of E3 ablated both IFN-*β* expression and NF-*κ*B activity in response to the dsDNA, poly(dA–dT). However, surprisingly, the ability of E3 to block poly(dA–dT) signalling was independent of the N terminus, whereas the dsRNA-binding domain was essential, suggesting that the Z-DNA-binding domain does not bind immunostimulatory dsDNA. This was confirmed by the failure of E3 to co-precipitate with biotinylated dsDNA, whereas the recruitment of several cytoplasmic DNA-binding proteins could be detected. Recently, AT-rich dsDNA was reported to be transcribed into 5′-triphosphate poly(A-U) RNA by RNA polymerase III, which then activates retinoic acid-inducible gene I (RIG-I). Consistent with this, RNA from poly(dA–dT) transfected cells induced IFN-*β* and expression of the E3 dsRNA-binding domain was sufficient to ablate this response. Given the well documented function of the E3 dsRNA-binding domain we propose that E3 blocks signalling in response to poly(dA–dT) by binding to transcribed poly(A-U) RNA preventing RIG-I activation. This report describes a DNA virus-encoded inhibitor of the RNA polymerase III-dsDNA-sensing pathway and extends our knowledge of E3 as a modulator of innate immunity.

## INTRODUCTION

*Vaccinia virus* (VACV) is the prototypical member of the genus *Orthopoxvirus* (OPV). As with all poxviruses, VACV is a large dsDNA virus that replicates within the cytoplasm of infected cells (Moss, 2007). This replication strategy results in the production of immunostimulatory nucleic acids that have the potential to activate the innate immune response via pattern-recognition receptors (PRRs). Not surprisingly, VACV therefore encodes many intracellular and extracellular modulators of innate immunity that serve to prevent the host from mounting an effective immune response to infection (for examples see Alcami & Smith, 1992; Carroll *et al.*, 1993; Chen *et al.*, 2008; Gedey *et al.*, 2006; Reading *et al.*, 2003; Symons *et al.*, 1995; for reviews see Perdiguero & Esteban, 2009; Seet *et al.* 2003).

Self and non-self cytoplasmic dsDNA induces the expression of type I interferon (IFN) and NF-*κ*B regulated genes independently of Toll-like receptor 9 (TLR9) (Ishii *et al.*, 2006). However, the signalling pathways involved in this process are poorly understood. The DNA-dependent activator of IFN regulatory factors, DAI (also known as ZBP1), was one candidate for the activation of IFN signalling in response to dsDNA in certain cell types (Takaoka *et al.*, 2007). DAI contains two Z-DNA-binding domains that bind dsDNA in a sequence-independent manner. Once bound to dsDNA, DAI is thought to recruit TANK-binding kinase 1 (TBK1) and IFN regulatory factor 3 (IRF3). IRF3 is then activated and translocates to the nucleus, resulting in the induction of type I IFN (Takaoka *et al.*, 2007). In addition, DAI also recruits receptor-interacting protein kinases, RIP1 and RIP3, through RIP homotypic interaction motif-dependent interactions, resulting in the activation of NF-*κ*B (Kaiser *et al.*, 2008). However, other dsDNA sensors must exist because murine embryonic fibroblasts (MEFs) lacking DAI still respond normally to intracellular dsDNA (Wang *et al.*, 2008). Indeed, recent work has identified absent in melanoma 2 (AIM2) (Burckstummer *et al.*, 2009; Fernandes-Alnemri *et al.*, 2009; Hornung *et al.*, 2009; Roberts *et al.*, 2009) and RNA polymerase III (Ablasser *et al.*, 2009; Chiu *et al.*, 2009) as two further dsDNA sensors.

As a consequence of its cytoplasmic replication it is probable that VACV will encode inhibitors of cytosolic dsDNA sensing, and one candidate is E3. E3 is an intracellular protein that is highly conserved among OPVs and is expressed early during infection localizing to both the cytoplasm and nucleus (Watson *et al.*, 1991; Yuwen *et al.*, 1993). E3 is important for VACV resistance to IFN, and can be split into distinct N- and C-terminal halves. The C terminus of E3 contains a dsRNA-binding domain that serves to sequester dsRNA produced during viral replication, thereby preventing the activation of protein kinase R (PKR) and RNase L, as well as, the activation of IRF3 (Chang & Jacobs, 1993; Chang *et al.*, 1992, 1995; Watson *et al.*, 1991; Xiang *et al.*, 2002). Consequently, the dsRNA-binding domain is important for the IFN resistant phenotype of VACV (Brandt & Jacobs, 2001; Chang *et al.*, 1995).

The E3 N terminus, on the other hand, contains a region with amino acid similarity to the Z-DNA-binding domain of DAI (Takaoka *et al.*, 2007). The biological significance of this similarity is poorly understood; however, the N terminus is required for full VACV virulence (Brandt *et al.*, 2005; Brandt & Jacobs, 2001). Interestingly, the Z-DNA-binding domain of DAI and another Z-DNA-binding protein, adenosine deaminase 1 (ADAR1), can substitute for the E3 N terminus in promoting VACV virulence (Kim *et al.*, 2003). Furthermore, mutation of residues in the putative Z-DNA-binding domain of E3 analogous to those involved in contacting Z-DNA in ADAR1 and DAI reduced VACV virulence (Kim *et al.*, 2003), suggesting that the E3 N terminus functions by binding dsDNA. Intriguingly, expression of full-length E3 was reported to reduce IFN-*β* expression in response to the dsDNA species poly(dA–dT) in MEFs (Wang *et al.*, 2008). However, the role of the N terminus in this phenotype was not investigated. These observations, coupled with reports that DAI functions as a PRR prompted us to investigate whether the E3 Z-DNA-binding domain antagonizes intracellular dsDNA PRRs by sequestering dsDNA.

## RESULTS

### Transfection of poly(dA–dT) induces IFN-*β* in a dose-dependent manner independently of TLR9

**Fig. 1.**
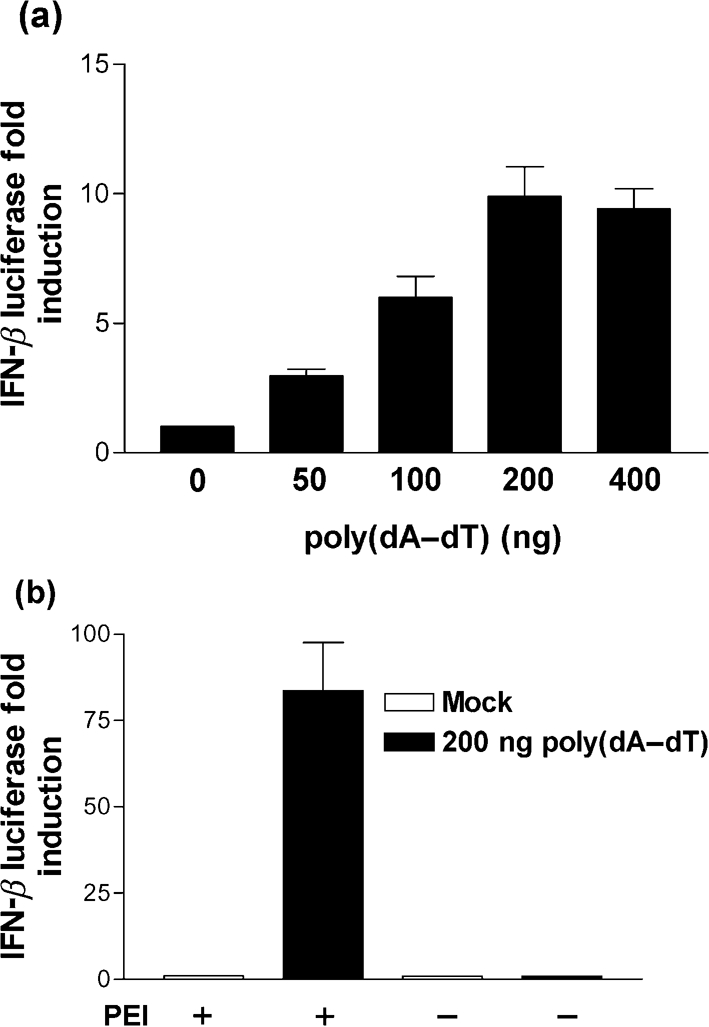
Poly(dA–dT) induces IFN-*β* reporter activity in a dose-dependent and TLR9-independent manner. 293T cells were co-transfected with pRL-TK and IFN-*β* reporter plasmids overnight. (a) The cells were then transfected with the indicated amounts of poly(dA–dT) for 24 h prior to the harvesting of cells for dual luciferase reporter assay. (b) 200 ng poly(dA–dT) was added to the cells in the presence, or absence, of the transfection reagent PEI, as indicated, and the cells then lysed 24 h later for dual luciferase reporter assay. Errors bars indicate the mean±sem.

The dsDNA, poly(dA–dT), is a potent inducer of type I IFN following the transfection of MEFs (Ishii *et al.*, 2006), and E3 can inhibit IFN-*β* expression in response to poly(dA–dT) (Marq *et al.*, 2009; Wang *et al.*, 2008). For consistency with previous reports we therefore used poly(dA–dT) in this study. Initially, a suitable reporter assay system in which the role of the E3 N terminus in blocking dsDNA signalling could be tested was developed by transfecting 293T cells with an IFN-*β* reporter and titrating increasing doses of poly(dA–dT). In accordance with previous reports, poly(dA–dT) induced IFN-*β* reporter activity in a dose-dependent manner (Fig. 1a).

293T cells express low levels of TLR9, which detects extracellular unmethylated CpG DNA (Hemmi *et al.*, 2000). Although, it seems unlikely that poly(dA–dT) would be a natural ligand for TLR9 it was necessary to demonstrate that induction of IFN-*β* by poly(dA–dT) was TLR9 independent. Accordingly, 293T cells were transfected with poly(dA–dT) or, alternatively, poly(dA–dT) was added to the culture medium in the absence of transfection reagent (polyethylenimine, PEI) and the effects upon IFN-*β* reporter activity were determined. Transfection of poly(dA–dT) led to a 75-fold activation of IFN-*β* reporter activity relative to the mock control. However, this effect was completely ablated when poly(dA–dT) was added to the culture medium in the absence of transfection reagent (Fig. 1b). These results, therefore, confirm that poly(dA–dT) is a potent stimulator of an intracellular dsDNA PRR.

### The E3 dsRNA-binding domain inhibits IFN-*β* expression in response to poly(dA–dT) independently of the E3 Z-DNA-binding domain

**Fig. 2.**
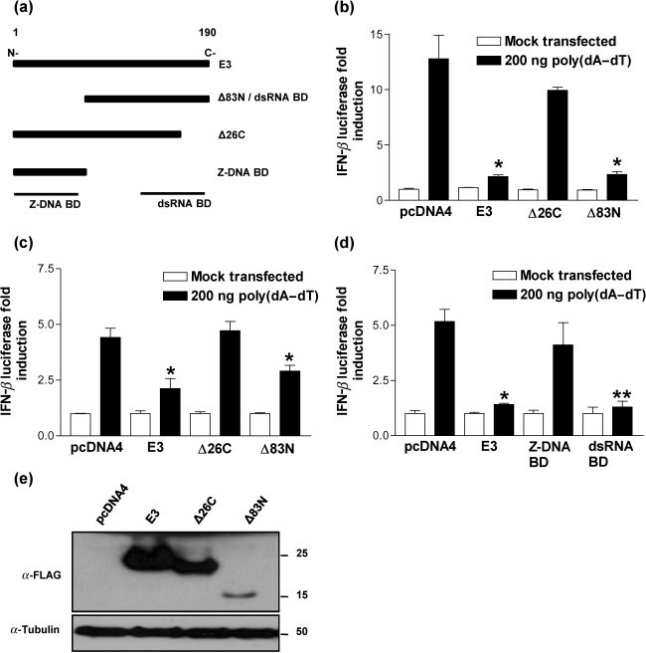
E3 inhibits IFN-*β* production in response to poly(dA–dT) via its C-terminal dsRNA-binding domain. (a) Schematic of full-length E3 and mutants in the C-terminal dsRNA-binding and N-terminal Z-DNA-binding domains. Amino acid positions are indicated at the top of full-length E3. (b) 293T and (c) HeLa cells were co-transfected with pRL-TK and IFN-*β* reporter plasmids together with pcDNA4, E3, Δ26C and Δ83N plasmids overnight. These cells were then transfected with 200 ng poly(dA–dT) and lysed for dual luciferase reporter assay after 24 h. (d) HeLa cells were co-transfected with pRL-TK and IFN-*β* reporter plasmids together with pcDNA4, E3, E3 dsRNA-binding domain and E3 Z-DNA-binding domain plasmids overnight and treated as described for (b) and (c). (e) 293T cells were transfected with pcDNA4, E3, Δ26C and Δ83N plasmids overnight. Cell lysates were then resolved by SDS-PAGE (12 % gel) and immunoblotted using anti-FLAG mAb (1 : 1000). Anti-*α*-tubulin mAb (1 : 5000) served as a protein loading control. The positions of molecular mass markers in kDa are indicated.

Next, the ability of E3 to block IFN-*β* reporter activity in response to the transfection of poly(dA–dT) was tested. 293T cells were co-transfected with the IFN-*β* reporter together with expression plasmids encoding N-terminally FLAG-tagged E3 and deletion mutants lacking either the N-terminal 83 aa (Δ83N) or the C-terminal 26 aa (Δ26C) (Fig. 2a). Transfection of poly(dA–dT) and empty vector control (pcDNA4) resulted in a 13-fold induction of IFN-*β* luciferase activity, and in agreement with earlier reports (Marq *et al.*, 2009; Wang *et al.*, 2008), expression of full-length E3 led to an almost complete ablation of IFN-*β* reporter activity. However, contrary to what was expected, Δ83N lacking the Z-DNA-binding domain was almost as efficient as full-length E3 in blocking IFN-*β*. In contrast, removal of the C-terminal 26 aa, which prevents E3 binding dsRNA (Chang & Jacobs, 1993), removed the inhibitory activity and was comparable to the empty vector control (Fig. 2b). Similar results were also obtained in HeLa cells confirming these effects were not cell type specific (Fig. 2c). Furthermore, the inability of the Z-DNA-binding domain to block poly(dA–dT) signalling was confirmed using a different set of vectors expressing either the Z-DNA-binding (aa 1–83) or dsRNA-binding (Δ83N, aa 84–190) domains of E3 (Fig. 2a, d) both of which are expressed at equivalent levels (Fig. 2e and data not shown). The failure of Δ26C to inhibit IFN-*β* reporter activity was not due to an absence of protein because immunoblotting with anti-FLAG monoclonal antibody (mAb) confirmed protein expression (Fig. 2e). Furthermore, despite being relatively unstable in comparison to full-length E3 and Δ26C, the Δ83N deletion mutant is still a potent inhibitor of poly(dA–dT) signalling.

**Fig. 3.**
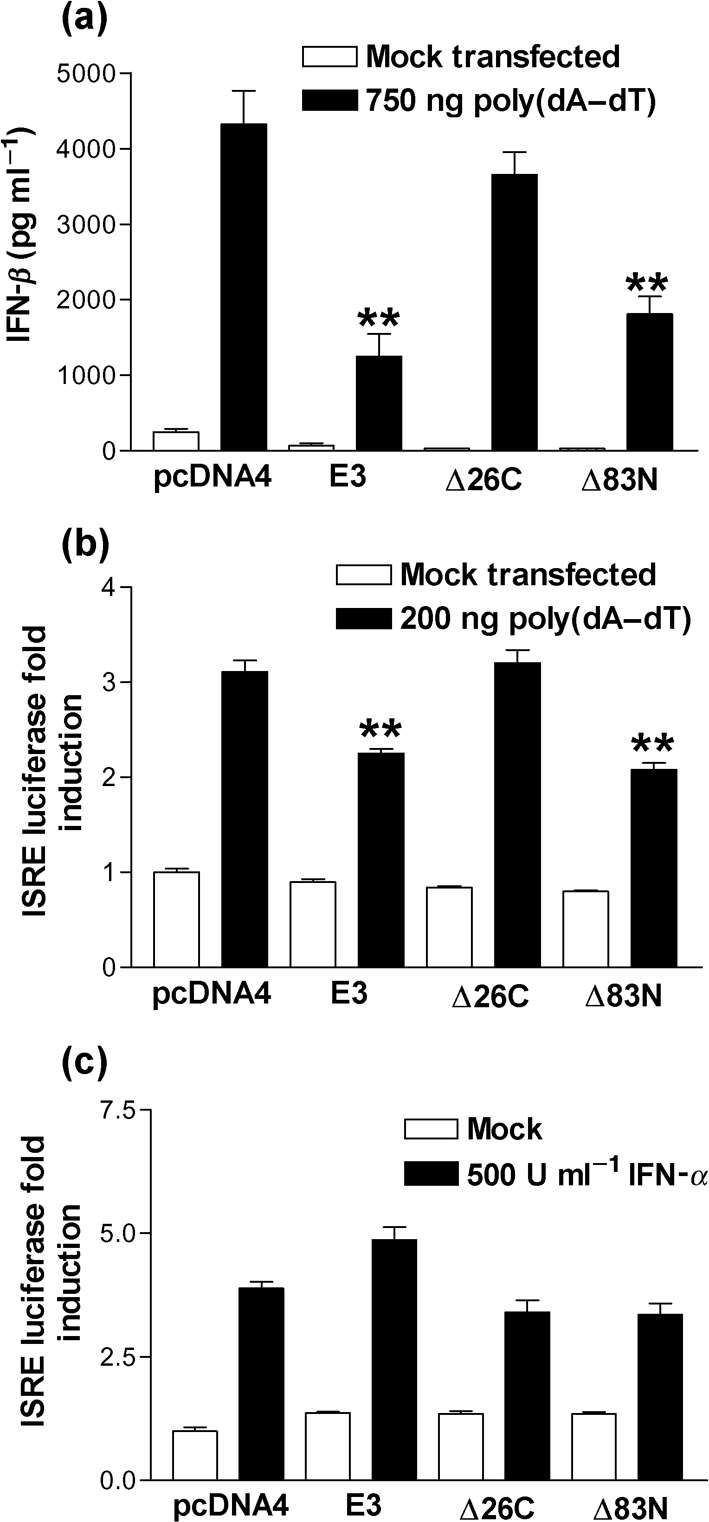
E3 inhibits the secretion of IFN-*β* and subsequent ISRE activation in response to poly(dA–dT). (a) 293T cells were transfected with pcDNA4, E3, Δ26C and Δ83N plasmids overnight. The cells were then transfected with 750 ng poly(dA–dT) for 24 h and IFN-*β* in the cell supernatant was measured by ELISA. (b, c) 293T cells were co-transfected with pRL-TK and ISRE reporter plasmids together with pcDNA4, E3, Δ26C and Δ83N plasmids overnight. The cells were then either (b) transfected with 200 ng poly(dA–dT) for 24 h or (c) stimulated with 500 U IFN-*α* ml^−1^ for 7 h prior to the harvesting of cells for dual luciferase reporter assay. Error bars indicate the mean±sem (*P<0.05, **P<0.01).

The ability of wild-type and mutant E3 to block IFN-*β* secretion from 293T cells induced by poly(dA–dT) was tested by ELISA. In agreement with the luciferase data transfection of poly(dA–dT) induced IFN-*β* secretion in those cells transfected with pcDNA4 and this was blocked following expression of either E3 or Δ83N (Fig. 3a). However, this inhibitory effect was lost following the expression of Δ26C, confirming that the dsRNA, but not Z-DNA-binding domain, is important for the inhibition of poly(dA–dT) signalling. The ability of E3 to block IFN-stimulated response element (ISRE) reporter activity in response to poly(dA–dT) was examined next. It was hypothesized that if the E3 dsRNA-binding domain was inhibiting IFN-*β* production in response to poly(dA–dT), an indirect inhibition of ISRE activity should be observed. In agreement with the ELISA data (Fig. 3a), transfection of poly(dA–dT) led to a modest threefold stimulation of ISRE activity, whereas both full-length E3 and Δ83N produced a statistically significant inhibition (Fig. 3b). This effect was completely ablated in those cells expressing E3 Δ26C, consistent with the IFN-*β* reporter data. Moreover, E3 was unable to inhibit ISRE reporter activity in response to IFN-*α* stimulation, confirming that the E3-mediated reduction in ISRE activity was not a consequence of E3 directly modulating the Jak/STAT signalling pathway (Fig. 3c).

**Fig. 4.**
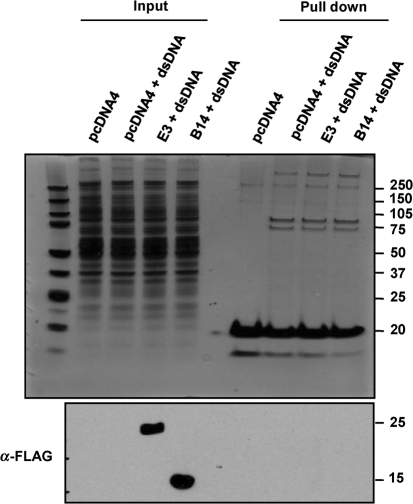
E3 does not bind cytoplasmic biotinylated dsDNA. 293T cells were transfected with plasmids expressing either FLAG-tagged B14 or E3 or an empty vector control (pcDNA4). The following day the cells were transfected with 20 μg biotinylated dsDNA and 4 h post-transfection the cells were lysed and the biotinylated dsDNA was pulled down from the cytoplasmic fraction using 100 μl streptavidin agarose. The pulled down fractions were then resolved on NuPAGE 4–12 % Bis-Tris pre-cast gels and then either silver stained (upper panel) or immunoblotted using anti-FLAG mAb (1 : 1000) (lower panel). The positions of molecular mass markers in kDa are indicated.

To extend these observations we tested whether E3 associated with cytoplasmic biotinylated dsDNA by co-precipitation and found no interaction by either immunoblotting or silver staining (Fig. 4). In contrast, the interaction of several cytoplasmic dsDNA-binding proteins with biotinylated DNA was detected and this was unaffected by the expression of E3 (Fig. 4 upper panel). 293T cells transfected with a plasmid expressing FLAG-tagged VACV protein B14 served as a negative control. The B14 protein binds IKK*β* and inhibits NF-*κ*B activation (Chen *et al.*, 2008) and would not be expected to bind dsDNA. In line with this, no binding of B14 to biotinylated dsDNA was detected (Fig. 4). Therefore, contrary to what was expected, the E3 dsRNA-binding domain is an inhibitor of the poly(dA–dT) signalling pathway, whilst the E3 Z-DNA-binding domain is entirely dispensable.

### The E3 dsRNA-binding domain inhibits NF-*κ*B activity in response to poly(dA–dT)

**Fig. 5.**
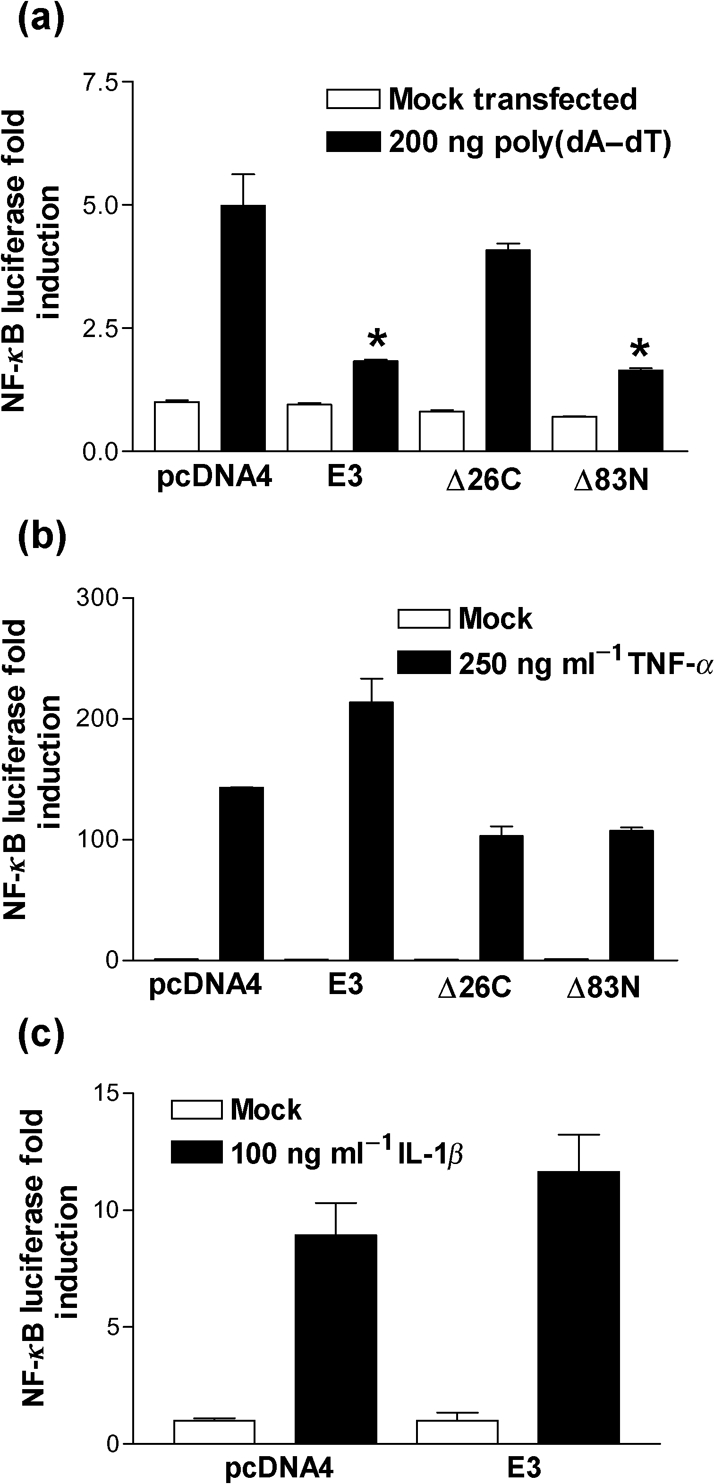
The E3 dsRNA-binding domain inhibits NF-*κ*B reporter activity in response to poly(dA–dT). 293T cells were co-transfected with pRL-TK and NF-*κ*B reporter plasmids together with pcDNA4, E3, Δ26C and Δ83N plasmids overnight. The cells were then either (a) transfected with 200 ng poly(dA–dT) for 24 h or stimulated with (b) 250 ng TNF-*α* ml^−1^ or (c) 100 ng IL-1*β* ml^−1^ for 7 h prior to the harvesting of cells for dual luciferase reporter assay. Error bars indicate the mean±sem (*P<0.05).

The transfection of dsDNA induces not only IFN-*β* expression but also NF-*κ*B activation (Kaiser *et al.*, 2008; Takaoka *et al.*, 2007) and, therefore, the ability of E3 to block NF-*κ*B reporter activity in response to poly(dA–dT) was investigated. Transfection of poly(dA–dT) resulted in a fivefold activation of luciferase activity in cells transfected with the empty vector control plasmid and this was blocked in cells expressing either full-length E3 or Δ83N. In contrast, Δ26C was not inhibitory (Fig. 5a). This is consistent with the E3 dsRNA-binding domain being crucial for E3 to block signalling in response to poly(dA–dT), whilst the Z-DNA-binding domain is not required (Figs 2 and 3).

Several pro-inflammatory signalling pathways converge on the IKK complex, resulting in the phosphorylation of I*κ*B*α* and the translocation of p65 and p50 homo- and heterodimers to the nucleus (Hayden & Ghosh, 2008). Since E3 can block NF-*κ*B activity in response to poly(dA–dT) it was important to determine whether E3 was a broad spectrum NF-*κ*B inhibitor or whether inhibition was specific to the poly(dA–dT)-sensing pathway. To address this, 293T cells were co-transfected with the NF-*κ*B reporter, and E3 expression plasmids, and then treated with tumour necrosis factor-alpha (TNF-*α*). Treatment of the control cells with TNF-*α* induced a strong induction of NF-*κ*B reporter activity. However, in contrast to poly(dA–dT) stimulation, neither full-length E3 nor Δ83N caused statistically significant repression of this activity (compare Fig. 5a against 5b). Similar results were obtained when using IL-1*β* to activate NF-*κ*B (Fig. 5c). These data confirm that E3 is not a broad spectrum NF-*κ*B inhibitor.

### E3 is an inhibitor of the RNA polymerase III cytoplasmic dsDNA signalling pathway

**Fig. 6.**
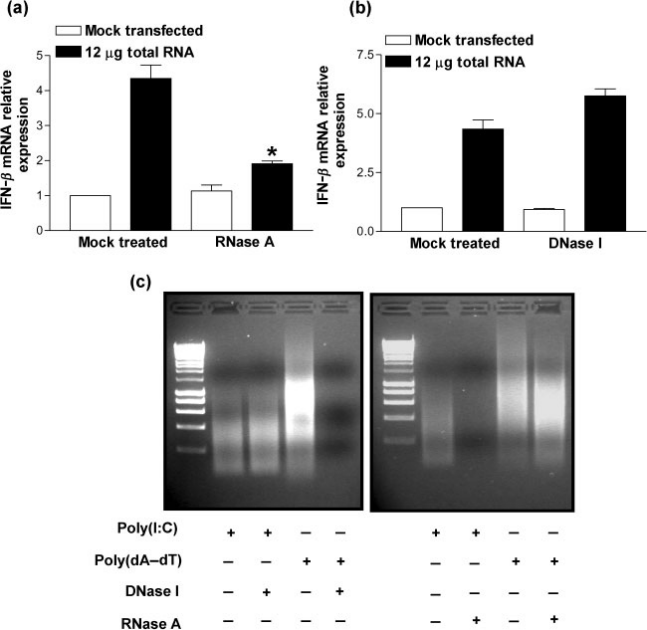
Transfection of total RNA harvested from poly(dA–dT) transfected cells results in IFN-*β* expression. 293T cells were transfected with 12 μg total RNA extracted from 293T cells that had been transfected with 12 μg poly(dA–dT) for 24 h. Induction of IFN-*β* mRNA was confirmed to be specific for an immunostimulatory RNA species by treating total RNA with either (a) 0.1 mg RNase A ml^−1^ or (b) 0.1 U amplification grade DNase I μl^−1^. Total RNA was then extracted 24 h post-transfection and real-time qPCR performed for IFN-*β*. These data were normalized against GAPDH mRNA levels. Error bars indicate the mean±sem (*P<0.05). (c) Cleavage of dsRNA or dsDNA by treatment with either RNase A or DNase I, respectively, was confirmed by treating poly(I : C) and poly(dA–dT) under the same conditions as for (a) and (b), respectively. The samples were then resolved on a 1 % agarose gel and stained using ethidium bromide.

Recently, RNA polymerase III was described as a novel cytoplasmic PRR for AT-rich dsDNA (Ablasser *et al.*, 2009; Chiu *et al.*, 2009). Specifically, RNA polymerase III transcribes poly(dA–dT), resulting in the production of 5′-triphosphate poly(A-U) RNA. This RNA then serves as a pathogen-associated molecular pattern (PAMP) for the PRR, retinoic acid-inducible gene I (RIG-I), resulting in IFN-*β* production (Yoneyama *et al.*, 2004). Consistent with these reports, transfection of total RNA harvested from poly(dA–dT) transfected cells resulted in IFN-*β* mRNA upregulation. Treatment of the RNA preparation with RNase A under low salt conditions, which results in the cleavage of dsRNA, ablated this induction (Fig. 6a), whereas treatment with DNase I did not (Fig. 6b). The specificity of the enzymic reactions was confirmed by treating the dsRNA analogue, poly(I : C), or the dsDNA, poly(dA–dT), with either RNase A or DNase I, respectively, under the same conditions as described above (Fig. 6c).

**Fig. 7.**
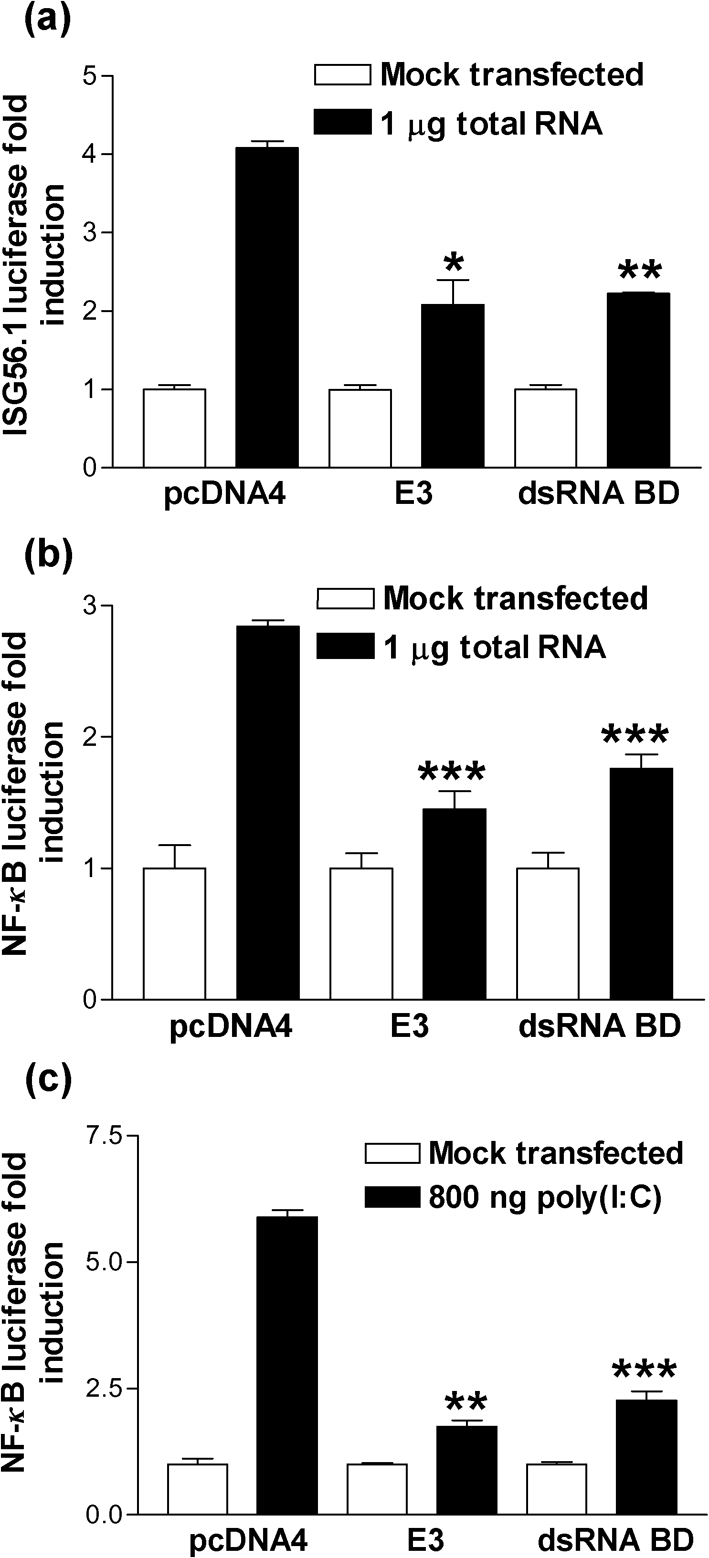
The E3 dsRNA-binding domain blocks IRF3 and NF-*κ*B activation in response to poly(dA–dT) transcribed RNA. 293T cells were co-transfected with pRL-TK and either (a) ISG56.1 or (b) NF-*κ*B reporter plasmids together with plasmids expressing either E3 or the E3 dsRNA-binding domain or the empty vector control (pcDNA4) overnight. The cells were then transfected with 1 μg total RNA extracted from 293Ts that had been transfected with 12 μg poly(dA–dT) for 24 h. At 24 h post-transfection, cells were lysed for dual luciferase reporter assay. (c) As a positive control 293T cells were co-transfected with the NF-*κ*B reporter plasmid as described for (b) and the following day were transfected with 800 ng poly(I : C) for 24 h prior to harvesting of cells for dual luciferase reporter assay. Error bars indicate the mean±sem (*P<0.05, **P<0.01, ***P<0.001).

The binding of 5′-triphosphate dsRNA by RIG-I results in the recruitment of the adaptor protein, IFN-*β* promoter stimulator 1 (IPS-1), which then acts as a scaffold for the activation of both IRF3 and NF-*κ*B (Kawai *et al.*, 2005). Indeed, transfection of the total RNA, which induced IFN-*β*, resulted in both ISG56.1 (Fig. 7a) and NF-*κ*B (Fig. 7b) stimulation in 293T cells. ISG56.1 is a transcriptional target for IRF3 and is, therefore, a good readout for IRF3-specific activation (Grandvaux *et al.*, 2002). However, this luciferase activity was ablated by E3, and the C-terminal dsRNA-binding domain was sufficient to mediate this inhibition (Fig. 7a, b). Furthermore, the level of luciferase activity obtained following transfection of the total RNA and subsequent inhibition by E3 was in line with that obtained when transfecting the synthetic dsRNA, poly(I : C), which is known to engage RIG-I (Fig. 7c). These results, therefore, support the conclusion that the immunostimulatory RNA engages the RIG-I signalling pathway and that E3 inhibits this process.

## DISCUSSION

The innate immune response represents the first line of defence against pathogenic micro-organisms. PRRs serve to detect conserved PAMPs. Once engaged PRRs signal via adaptor proteins to activate NF-*κ*B and IRF3, resulting in the secretion of type I IFN and pro-inflammatory cytokines and chemokines (Randall & Goodbourn, 2008). The VACV E3 protein is a well known inhibitor of dsRNA PRRs and antiviral molecules. It was proposed that E3 may also function as an inhibitor of intracellular dsDNA PRRs by sequestering dsDNA via the N-terminal Z-DNA-binding domain (Wang *et al.*, 2008). However, this hypothesis is based on amino acid similarity to the cytoplasmic dsDNA sensor, DAI, and has never formally been demonstrated. In agreement with recent reports we confirm that E3 is a potent inhibitor of type I IFN expression in response to the dsDNA, poly(dA–dT) (Marq *et al.*, 2009; Wang *et al.*, 2008). Using E3 deletion mutants we show that the assumption that this inhibition is mediated via the Z-DNA-binding domain is untrue. Indeed, the ability of E3 to block signalling in response to poly(dA–dT) maps entirely to the C-terminal dsRNA-binding domain. The inability of the E3 Z-DNA-binding domain to bind cytoplasmic dsDNA is also supported by pull-down assays in which biotinylated dsDNA could recruit other DNA-binding proteins, but not E3. This indicates that the virulence promoting function of the E3 N terminus is not due to binding of cytoplasmic dsDNA. Given the amino acid similarity of the E3 and DAI Z-DNA-binding domains these results also question whether the primary function of DAI is as a cytoplasmic PRR.

Whilst this study was ongoing, RNA polymerase III was described as a novel cytoplasmic PRR for dsDNA. RNA polymerase III was found to transcribe AT-rich DNA, resulting in the production of 5′-triphosphate poly(A-U) RNA, which then served as a PAMP for RIG-I (Ablasser *et al.*, 2009; Chiu *et al.*, 2009). These reports, therefore, perfectly explain both the observations from our group, and others, that the E3 dsRNA-binding domain can inhibit poly(dA–dT) signalling (Marq *et al.*, 2009). Indeed, we have confirmed that the transfection of poly(dA–dT) results in the production of an immunostimulatory RNA, which activates both IRF3 and NF-*κ*B, thereby inducing type I IFN. Furthermore, expression of the E3 dsRNA-binding domain is sufficient to antagonize this response, suggesting that E3 is specifically binding the immunostimulatory RNA preventing activation of divergent signalling pathways downstream of the RIG-I receptor complex. In addition to the RNA polymerase III-sensing pathway, RIG-I may bind directly to dsDNA (Choi *et al.*, 2009). However, we found no evidence to support E3 associating with dsDNA, ruling out the possibility that the E3 dsRNA-binding domain associates directly with DNA. We conclude that E3 is a novel inhibitor of the RNA polymerase III AT-rich DNA-sensing pathway and that this is mediated via the well characterized function of E3 binding to dsRNA. This might be important for VACV replication since the VACV genome is AT-rich and, therefore, one would envisage VACV being particularly sensitive to sensing via RNA polymerase III (Moss, 2007). This is difficult to test directly, however, due to the essential function of RNA polymerase III within cells.

Whilst data presented in this report helps further clarify why VACV lacking the C terminus of the E3 protein presents with such an avirulent phenotype, the function of the N terminus in virulence remains unclear (Brandt & Jacobs, 2001; Chang *et al.*, 1995). Although our studies suggest that E3 does not bind immunostimulatory dsDNA, which is likely to reside within a right-handed B-DNA conformation, we cannot rule out that the E3 N terminus associates with left-handed Z-DNA. The formation of Z-DNA occurs when RNA polymerase II moves along DNA, resulting in negative supercoiling behind the transcription complex. Furthermore, sequences that favour the formation of Z-DNA may be a common feature of most gene transcription start sites (Rich & Zhang, 2003). It is, therefore, interesting to speculate that the importance of the E3 N terminus and other Z-DNA-binding proteins might lie in regulating cellular transcription. Indeed, E3 has been reported to upregulate the transcription of certain genes (Kwon & Rich, 2005), although, this remains controversial (Langland *et al.*, 2006). The mechanism by which the E3 N terminus promotes virulence remains unknown.

In summary, our results extend our knowledge of E3 as an inhibitor of innate immunity and provide an example of a dsDNA virus encoding an inhibitor of the recently described RNA polymerase III-dependent sensing of dsDNA in innate immunity.

## METHODS

### Cell culture.

Human embryonic kidney 293T and HeLa cells were cultured in Dulbecco's modified Eagle's medium (DMEM; Gibco) supplemented with 10 % fetal bovine serum, 100 U penicillin ml^−1^ and 100 μg streptomycin ml^−1^ and 2 mM l-glutamine (Sigma-Aldrich).

### Plasmids.

N-terminal FLAG-tagged E3, Δ26C and Δ83N were generated by PCR using the *E3L* gene from VACV strain Western Reserve (WR) as template. The PCR products were then cloned into the multiple cloning site of pcDNA4 TO (Invitrogen). The IFN-*β*, ISRE, IFN-stimulated gene 56.1 (IRF3) and NF-*κ*B reporters, which drive firefly luciferase expression, and the *Renilla* luciferase control plasmid (pRL-TK) have been described previously (Chen *et al.*, 2008).

### Reporter assays.

293T cells were seeded into 96-well tissue culture plates overnight prior to transfection with 70 ng of the indicated reporter plasmids, 10 ng pRL-TK and 70 ng of the appropriate E3 expression plasmid or empty vector control (pcDNA4) as indicated using PEI (Park Scientific). Following incubation overnight cells were stimulated as follows. For dsDNA stimulation, 293T cells were transfected with the indicated amounts of poly(dA–dT) (Sigma-Aldrich) for 24 h. For IFN-*α*, TNF-*α* and IL-1*β* (Peprotech) stimulations the cells were treated at the indicated concentrations for 7 h. Once the stimulation was complete cell lysates were harvested using passive lysis buffer and dual luciferase reporter assays were performed following the manufacturer's instructions (Promega). Relative luciferase activity was determined by normalizing the firefly luciferase data against the respective *Renilla* luciferase control. In all cases, data are from one of two to four independent experiments with similar qualitative results. Data from experiments performed in triplicate are expressed as means±sem. Statistical analysis was performed by an F-test to determine equal or unequal variance followed by a Student's *t*-test where appropriate.

### ELISA.

Levels of IFN-*β* in the supernatant of 293T cells were measured using a human IFN-*β*-specific ELISA kit (PBL Biomedical Laboratories) following the manufacturer's instructions.

### DNA pull-down assay and immunoblotting.

DNA pull-down assays were performed by transfecting 293T cells with biotinylated dsDNA (Integrated DNA Technologies) using PEI. Following 4 h incubation, cells were lysed on ice using buffer containing 10 mM Tris/HCl, pH 8, 0.1 % NP-40, 10 mM MgCl_2_ and the cytoplasmic fraction was isolated. This was incubated with streptavidin agarose for 2 h and the beads were then washed three times with chilled PBS prior to analysis by both immunoblotting and silver staining (Invitrogen).

Immunoblotting was performed as described previously (Chen *et al.*, 2008) using mouse anti-FLAG (Sigma-Aldrich) and mouse anti-*α*-tubulin primary mAbs (Upstate).

### RNA isolation and enzyme treatment of nucleic acids.

Total RNA was isolated using TRIzol reagent (Invitrogen) following the manufacturer's instructions. Nucleic acids were treated either with RNase A using the conditions described previously (Chiu *et al.*, 2009) or with amplification grade DNase I following the manufacturer's instructions (Invitrogen).

### Real-time qPCR.

RNA was reverse transcribed using Superscript III Reverse Transcriptase (Invitrogen) following the manufacturer's instructions. Real-time qPCR was performed using FAST SYBR green master mix (Applied Biosystems) on a 7900HT thermocycler (Applied Biosystems) and data were analysed using the relative quantification manager software. Real-time qPCR for GAPDH was used to normalize all data.
